# Intracranial lesion yield and downstream management after mild traumatic brain injury in emergency department patients receiving antiplatelet therapy: a two-center retrospective cohort study

**DOI:** 10.1186/s12873-026-01684-8

**Published:** 2026-07-23

**Authors:** Fatima Di Padova, Tifany Sekulski, Dounia Hamdouche, Romain Jouffroy, Vincent Garrouste

**Affiliations:** 1 Emergency Department, Orléans University, Orléans University Hospital, 14 Avenue de l’hôpital, Orléans, 45100 France; 2 Sports Medicine Department, Orléans University, Orléans University Hospital, Orléans, 45100 France; 3 LI²RSO, Orléans University, Orléans University Hospital, Orléans, 45100 France

**Keywords:** Mild traumatic brain injury, Antiplatelet therapy, Intracranial lesion, Head computed tomography, Emergency department

## Abstract

**Background:**

Mild traumatic brain injury (mTBI) in patients receiving antiplatelet therapy is a frequent emergency department presentation. We aimed to describe the prevalence of traumatic intracranial lesions on the initial head computed tomography (CT) scan and the downstream in-hospital management of antiplatelet-treated adults with mTBI.

**Methods:**

We conducted a retrospective two-center cohort study in the emergency departments of Tours and Orléans University Hospitals, France. Adults receiving antiplatelet therapy who presented with mTBI (Glasgow Coma Scale score 13–15) and underwent an initial head CT scan were included. The primary outcome was the presence of any traumatic intracranial lesion on the initial CT scan. Secondary outcomes included downstream management during the index episode. An exploratory multivariable logistic regression analysis was performed using pre-CT variables.

**Results:**

A total of 1,912 patients were included; mean age was 80.8 ± 12.1 years and 52.1% were women. Traumatic intracranial lesions were identified in 282 patients (14.7%). Among patients with lesions, 232 (82.3%) underwent hospital admission or 24-hour emergency department observation, 16 (5.7%) required neurosurgical management, and 104 (36.9%) underwent repeat brain imaging. In exploratory multivariable analysis, falls above standing height or high-energy falls (adjusted odds ratio [aOR] 2.84, 95% confidence interval [CI] 1.95–4.14), other trauma mechanisms (aOR 1.82, 95% CI 1.30–2.54), motor deficit at presentation (aOR 3.71, 95% CI 1.95–6.81), facial fracture (aOR 2.25, 95% CI 1.51–3.31), and temporal impact (aOR 1.94, 95% CI 1.29–2.85) were independently associated with traumatic intracranial lesions.

**Conclusions:**

In antiplatelet-treated adults with mTBI, traumatic intracranial lesions were detected in approximately one in seven patients on the initial CT scan, whereas neurosurgical intervention remained uncommon. Trauma-related clinical features appeared more informative than age alone for lesion detection.

## Background

Mild traumatic brain injury (mTBI) is one of the most common reasons for emergency department (ED) consultation. Although only a minority of patients with mTBI ultimately have traumatic intracranial hemorrhage, diagnostic uncertainty remains substantial, particularly in older adults, in whom falls and antithrombotic treatments are frequent [1–5].

Clinical decision rules such as the Canadian CT Head Rule have structured the initial assessment of minor head injury for more than two decades [1]. However, their application to contemporary older ED populations receiving antithrombotic therapy remains challenging. In its 2023 clinical policy, the American College of Emergency Physicians recommended use of the Canadian CT Head Rule in adults with minor head injury in general, while also stating that current decision tools should not be used to reliably exclude the need for head CT in patients receiving anticoagulant or antiplatelet therapy other than aspirin [3]. Similarly, recent French and UK guidance continue to support a cautious imaging strategy in at-risk patients with mTBI [2, 4].

The specific impact of pre-injury antiplatelet therapy on the risk of intracranial bleeding after mTBI remains debated. In a large prospective multicenter study, Probst et al. reported a higher prevalence of significant intracranial injury in patients receiving combination aspirin and clopidogrel, whereas aspirin alone was not associated with a clearly increased risk [6]. In parallel, two meta-analyses found that pre-injury antiplatelet therapy was associated with a higher risk of traumatic intracranial hemorrhage after head injury, although the magnitude and clinical implications of this excess risk varied across studies [7, 8]. More recently, Mathieu et al. suggested that the subgroup at highest risk of adverse outcomes may be patients receiving dual antiplatelet therapy rather than single-agent therapy [9].

Questions also remain after the initial CT scan. Delayed intracranial bleeding appears uncommon overall after a negative initial CT in clinically stable patients receiving antiplatelet therapy, and routine repeated CT imaging may not be justified in patients on single-agent therapy [10–13]. At the same time, recent prospective work has suggested that the Canadian CT Head Rule might be applicable in selected patients on anticoagulant or antiplatelet therapy with a Glasgow Coma Scale score of 15, although this finding requires external confirmation before it can influence practice [14]. More broadly, recent decision-rule work in older adults after ground-level falls has highlighted the potential value of objective bedside features such as neurologic examination, amnesia, and frailty for identifying lower-risk patients [15, 16].

In this context, better descriptive data are needed on what is actually found on initial head CT in ED patients with mTBI receiving antiplatelet therapy, and on how often these findings lead to clinically meaningful changes in management. We therefore conducted a two-center retrospective cohort study to describe the prevalence and pattern of intracranial lesions, as well as downstream in-hospital management, in adult patients receiving antiplatelet therapy who presented to the ED with mild traumatic brain injury.

## Methods

### Study design and setting

We conducted a retrospective two-center cohort study in the emergency departments of Orléans and Tours University Hospitals, France. The primary objective was to describe the diagnostic yield of the initial head computed tomography (CT) scan and the downstream in-hospital management of adult patients receiving antiplatelet therapy who presented with mild traumatic brain injury (mTBI). The study period extended from April 2012 to December 2022 at Tours University Hospital and from January 2016 to December 2022 at Orléans University Hospital. The study design and initial data collection procedures were based on a previously assembled bicentric cohort.

### Study size

The study sample consisted of all eligible patients identified during the study period in both participating centers. No formal sample size calculation was performed because of the retrospective design.

### Participants

Eligible participants were adults aged 18 years or older receiving antiplatelet therapy, regardless of agent or dose, who presented to the emergency department for mild traumatic brain injury, defined by a Glasgow Coma Scale score of 13 to 15 at initial assessment. All included patients underwent an initial head CT scan during the index emergency department visit.

Although mild traumatic brain injury is not defined solely by the Glasgow Coma Scale, this retrospective study used an operational definition based on routine emergency department documentation: head trauma with an initial Glasgow Coma Scale score of 13 to 15. Symptoms such as loss of consciousness, amnesia, vomiting, or headache were not used as inclusion criteria because retrospective ascertainment was considered insufficiently reliable.

Patients were excluded if they had known coagulopathy, concomitant anticoagulant therapy, previously known neurological abnormalities interfering with interpretation of the initial examination, signs of moderate or severe traumatic brain injury, secondary clinical deterioration with a decrease in Glasgow Coma Scale score, or unavailable imaging data. Each patient was included only once in the study.

### Case identification and data sources

Potentially eligible patients were identified retrospectively from electronic medical records in each participating center using routine-care data extraction procedures specific to each hospital. At Tours University Hospital, patients were screened using emergency department admission diagnoses related to head trauma together with documentation of a Glasgow Coma Scale score between 13 and 15. At Orléans University Hospital, patients were identified using a prespecified list of ICD-10 codes related to head trauma and intracranial injury. Detailed chart review was then performed to confirm eligibility and extract study variables.

Data collection was performed by two emergency medicine residents using a standardized chart review process and was validated by two senior emergency physicians. When a clinical variable was not documented in the medical record, it was considered missing and coded as not available.

During the study period, downstream management was not governed by a unified prospective protocol across the two participating centers. Decisions regarding hospital admission or 24-hour emergency department observation, repeat brain imaging, corticosteroid therapy, and other lesion-related management measures were left to the treating team according to radiological findings, clinical course, and, when relevant, specialist advice. Platelet transfusion was not systematically captured in the retrospective dataset and was therefore not analyzed.

### Collected variables

The following variables were collected from medical records: age, sex, antiplatelet regimen, cardiovascular and neurological comorbidities, trauma mechanism, associated traumatic findings, point of impact, selected neurological findings at presentation, blood pressure category, prior cerebrovascular or neurosurgical history, CT findings, and in-hospital management variables during the index episode. Study center was recorded as Tours or Orléans.

For exploratory regression analyses, only variables available before interpretation of the initial CT scan were considered candidate predictors.

### Outcome measures

The primary outcome was the presence of any traumatic intracranial lesion on the initial head CT performed during the index emergency department visit. Intracranial lesions were defined according to the final radiology report drafted by the radiology resident and reviewed by a senior radiologist. These lesions included subdural hematoma, epidural hematoma, traumatic subarachnoid hemorrhage, intraparenchymal hemorrhage, intraventricular hemorrhage, petechial hemorrhage, or other traumatic intracranial bleeding. Multiple lesion subtypes could be recorded in the same patient; therefore, lesion subtype frequencies were described as non-mutually exclusive categories.

Secondary outcomes were descriptive and included downstream management during the index episode among patients with CT-detected intracranial lesions, namely hospital admission or 24-hour emergency department observation, neurosurgical management, corticosteroid therapy, modification or interruption of antiplatelet therapy, repeat brain imaging, and planned outpatient follow-up. Because of limited clinical interpretability and retrospective ascertainment, secondary complications were not retained as a study endpoint in the present analysis. Additional descriptive analyses explored hospital admission or 24-hour emergency department observation and repeat brain imaging in patients without traumatic intracranial lesions on the initial CT scan.

### Quantitative variables

Age was analyzed as a continuous variable. For exploratory multivariable analysis, trauma mechanism was grouped into three clinically meaningful categories to improve model stability: fall from standing height, fall above standing height or high-energy fall, and other mechanisms.

### Statistical analysis

The statistical analysis was primarily descriptive. Categorical variables were summarized as counts and percentages, and continuous variables as mean with standard deviation or median with interquartile range, as appropriate according to their distribution. Patients with and without traumatic intracranial lesions on the initial CT scan were compared using Fisher’s exact test for categorical variables and Student’s t test or the Mann–Whitney U test for continuous variables, as appropriate. All tests were two-sided, and a p value < 0.05 was considered statistically significant.

An exploratory multivariable logistic regression analysis was performed as a secondary analysis to identify pre-CT factors independently associated with traumatic intracranial lesions on the initial CT scan. Candidate predictors were selected a priori on clinical grounds and from previous literature, and included age, sex, trauma mechanism, motor deficit at presentation, facial fracture, and temporal point of impact. Results were reported as adjusted odds ratios with 95% confidence intervals. No automated stepwise selection procedure was used. Missing data were handled using complete-case analysis for regression models, and the number of available observations was reported when relevant. A sensitivity analysis additionally adjusted for dual versus single antiplatelet therapy.

All analyses were performed using R software (R Foundation for Statistical Computing, Vienna, Austria), version 4.5.3 [17].

### Reporting and ethics

The study was reported according to the STROBE recommendations for observational studies [18]. This retrospective study was conducted in accordance with the Declaration of Helsinki and the French regulatory framework for “recherches n’impliquant pas la personne humaine” (RNIPH) based on secondary routine-care data. Patients were informed about the secondary use of their data and could oppose participation. Data were pseudonymized before analysis.

## Results

### Study population

**Fig. 1:**
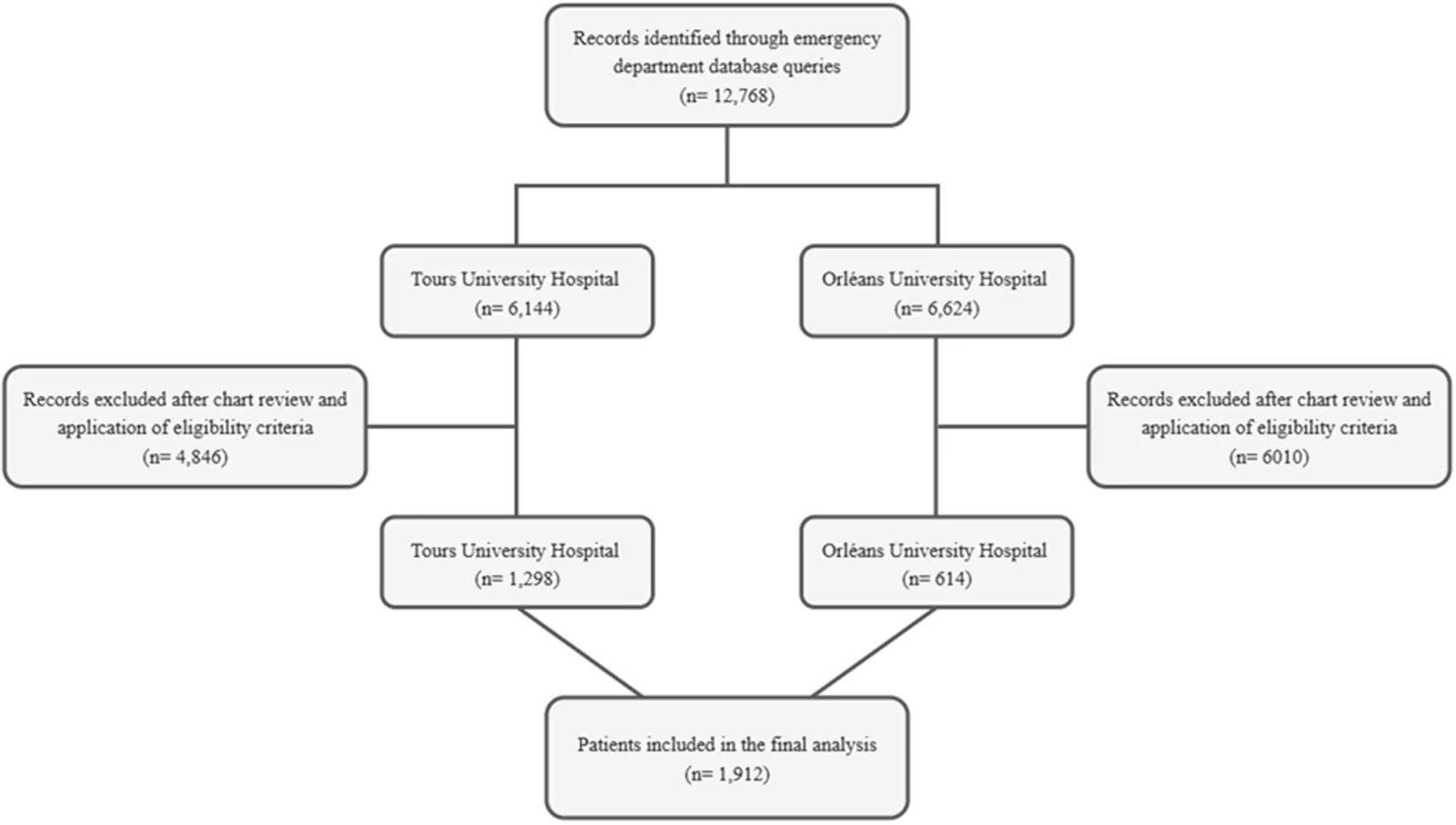
Flowchart

A total of 12,768 potentially eligible records were identified across the two participating centers during the study period. After chart review and application of the eligibility criteria, 1,912 patients were included in the final analysis, including 1,298 from Tours University Hospital and 614 from Orléans University Hospital (Fig. 1).

The cohort was elderly, with a mean age of 80.8 ± 12.1 years, and 996 patients (52.1%) were women (Table 1). Aspirin 75 mg was the most frequently documented antiplatelet agent, and falls from standing height were the predominant trauma mechanism (1,420/1,912, 74.3%).

**Table 1:** Baseline characteristics of the study cohort

Variable	Overall cohort (*n* = 1,912)^1^
Age, years	80.8 ± 12.1
Female sex	996 (52%)
Aspirin 75 mg	1,254 (66%)
Aspirin 160 mg	326 (17%)
Aspirin 300 mg	21 (1.1%)
Clopidogrel	349 (18%)
Ticagrelor	23 (1.2%)
Other antiplatelet agents	98 (5.1%)
Dual antiplatelet therapy	159 (8.3%)
Hypertension	1,543 (81%)
Diabetes	468 (24%)
Dyslipidemia	1,149 (60%)
Chronic kidney disease	89 (4.7%)
Current smoking	176 (9.2%)
Alcohol use	181 (9.5%)
Trauma mechanism	
Fall from standing height	1,420 (74%)
Fall above standing height / high-energy fall	187 (9.8%)
Other mechanisms	305 (16%)
Repeated falls	120 (6.3%)
Motor deficit at presentation	46 (2.4%)
Other fracture	186 (9.7%)
Facial fracture	162 (8.5%)
Associated wound	1,010 (53%)
Occipital impact	599 (31%)
Frontal impact	1,090 (57%)
Parietal impact	240 (13%)
Vertex impact	62 (3.2%)
Temporal impact	158 (8.3%)
History of stroke/bleeding/neurosurgery	623 (33%)
Prior cranial anatomical changes	26 (1.4%)

^1^Values are presented as mean ± standard deviation or n (%). Antiplatelet categories were not mutually exclusive because patients receiving dual antiplatelet therapy could contribute to both molecule-specific rows and the dual antiplatelet therapy row

### Initial CT findings

Among the 282 patients with traumatic intracranial lesions, subdural hematoma was the most frequent lesion subtype (57.0%), followed by traumatic subarachnoid hemorrhage (45.7%) and intraparenchymal hemorrhage (20.9%). Petechial hemorrhage was less frequent (11.0%), whereas epidural hematoma (1.4%) and intraventricular hemorrhage (0.7%) were uncommon. In unadjusted analyses (Table 2), patients with traumatic intracranial lesions were slightly younger than those without lesions (median age 82 [IQR 73–88] vs. 84 [75–90] years, *p* = 0.005) and were less often women (43% vs. 54%, *p* = 0.002). Dual antiplatelet therapy tended to be more frequent among patients with lesions, although this association did not reach conventional statistical significance (11% vs. 7.9%, *p* = 0.08).

**Table 2:** Comparison of patients with and without traumatic intracranial lesions on the initial head CT scan

Variable	No traumatic intracranial lesion (*n* = 1,630)^1^	Traumatic intracranial lesion (*n* = 282)^1^	*p*-value^2^
Age, years	84.0 [75–90]	82.0 [73–88]	0.005
Female sex	874 (54%)	122 (43%)	0.01
Antiplatelet regimen^3^			
Aspirin 75 mg	1,063 (65%)	191 (68%)	0.46
Aspirin 160 mg	278 (17%)	48 (17%)	> 0.99
Aspirin 300 mg	19 (1.2%)	2 (0.7%)	0.76
Clopidogrel	294 (18%)	55 (20%)	0.56
Ticagrelor	19 (1.2%)	4 (1.4%)	0.77
Other antiplatelet agents	85 (5.2%)	13 (4.6%)	0.77
Dual antiplatelet therapy	128 (7.9%)	31 (11%)	0.08
Hypertension	1,321 (81%)	222 (79%)	0.37
Diabetes	390 (24%)	78 (28%)	0.18
Dyslipidemia	979 (60%)	170 (60%)	> 0.99
Chronic kidney disease	77 (4.7%)	12 (4.3%)	0.88
Current smoking	140 (8.6%)	36 (13%)	0.03
Alcohol use	153 (9.4%)	28 (9.9%)	0.74
Trauma mechanism			< 0.001
Fall from standing height	1,254 (77%)	166 (59%)	
Fall above standing height / high-energy fall	133 (8.2%)	54 (19%)	
Other mechanisms	243 (15%)	62 (22%)	
Repeated falls	95 (5.8%)	25 (8.9%)	0.06
Motor deficit at presentation	28 (1.7%)	18 (6.4%)	< 0.001
Other fracture	149 (9.1%)	37 (13%)	0.04
Facial fracture	121 (7.4%)	41 (15%)	< 0.001
Associated wound	859 (53%)	151 (54%)	0.80
Occipital impact	504 (31%)	95 (34%)	0.37
Frontal impact	938 (58%)	152 (54%)	0.27
Parietal impact	190 (12%)	50 (18%)	0.01
Vertex impact	54 (3.3%)	8 (2.8%)	0.86
Temporal impact	120 (7.4%)	38 (13%)	< 0.001
History of stroke/bleeding/neurosurgery	530 (33%)	93 (33%)	0.89
Prior cranial anatomical changes	21 (1.3%)	5 (1.8%)	0.57

^1^Values are presented as median [interquartile range] or n (%). Categorical variables were compared using Fisher’s exact test and continuous variables using the Mann–Whitney U test, as appropriate. Antiplatelet categories were not mutually exclusive

^2^Wilcoxon rank sum test; Fisher’s exact test

^3^Antiplatelet categories were not mutually exclusive

Trauma-related characteristics differed substantially between groups. Compared with patients without lesions, those with traumatic intracranial lesions less frequently had falls from standing height (59% vs. 77%) and more frequently had falls above standing height or high-energy falls (19% vs. 8.2%) and other trauma mechanisms (22% vs. 15%) (overall *p* < 0.001). Road traffic collisions were also more frequent among patients with lesions (8.5% vs. 4.1%, *p* = 0.003). In addition, motor deficit at presentation (6.4% vs. 1.7%, *p* < 0.001), other fractures (13% vs. 9.1%, *p* = 0.049), facial fractures (15% vs. 7.4%, *p* < 0.001), parietal impact (18% vs. 12%, *p* = 0.006), and temporal impact (13% vs. 7.4%, *p* = 0.001) were more common in patients with traumatic intracranial lesions. Current smoking was also more frequent in the lesion group (13% vs. 8.6%, *p* = 0.033).

### Downstream management among patients with traumatic intracranial lesions

Among the 282 patients with traumatic intracranial lesions, 232 (82.3%) underwent hospital admission or 24-hour emergency department observation (Table 3), 16 (5.7%) required neurosurgical management, and 30 (10.6%) received corticosteroid therapy. Repeat brain imaging was performed in 104 patients (36.9%), and planned outpatient follow-up was reported in 35 (12.4%). Regarding antiplatelet therapy management during the index episode, treatment was discontinued in 84 patients (29.8%), temporarily suspended in 98 (34.8%), reduced in 14 (5.0%), and re-evaluated but left unchanged in 8 (2.8%).

**Table 3:** Downstream management among patients with traumatic intracranial lesions

Variable	Patients with traumatic intracranial lesion (*n* = 282)^1^
Hospital admission or 24-hour emergency department observation	232 (82.3%)
Corticosteroid therapy	30 (10.6%)
Neurosurgical management	16 (5.7%)
Antiplatelet therapy discontinued	84 (29.8%)
Antiplatelet therapy re-evaluated unchanged	8 (2.8%)
Antiplatelet therapy reduced	14 (5.0%)
Temporary suspension of antiplatelet therapy	98 (34.8%)
Repeat brain imaging	104 (36.9%)
Planned outpatient follow-up	35 (12.4%)

^1^Values are presented as n (%)

Among patients without traumatic intracranial lesions on the initial CT scan, 30 (1.8%) underwent hospital admission or 24-hour emergency department observation and 2 (0.1%) underwent repeat brain imaging.

### Exploratory multivariable analysis

In the prespecified exploratory multivariable logistic regression analysis, age was not independently associated with traumatic intracranial lesions on the initial CT scan (adjusted odds ratio [aOR] per year 0.996, *p* = 0.49). Female sex showed a non-significant trend toward lower odds of traumatic intracranial lesions (aOR 0.77, *p* = 0.055).

Compared with falls from standing height, falls above standing height or high-energy falls were independently associated with higher odds of traumatic intracranial lesions (aOR 2.84, 95% CI 1.95–4.14; *p* < 0.001), as were other trauma mechanisms (aOR 1.82, 95% CI 1.30–2.54; *p* < 0.001). Motor deficit at presentation (aOR 3.71, 95% CI 1.95–6.81; *p* < 0.001), facial fracture (aOR 2.25, 95% CI 1.51–3.31; *p* < 0.001), and temporal impact (aOR 1.94, 95% CI 1.29–2.85; *p* = 0.001) were also independently associated with traumatic intracranial lesions.

In the sensitivity analysis additionally adjusting for dual versus single antiplatelet therapy, the main associations remained materially unchanged. Dual antiplatelet therapy was associated with a borderline increase in the odds of traumatic intracranial lesions (aOR 1.54, 95% CI 0.98–2.39; *p* = 0.053), while higher-risk trauma mechanisms, motor deficit, facial fracture, and temporal impact remained independently associated with the outcome.

## Discussion

In this bicentric cohort of 1,912 adults receiving antiplatelet therapy and presenting to the ED with mTBI, traumatic intracranial lesions were identified on the initial head CT scan in 14.7% of patients. Among patients with CT-detected lesions, most underwent hospital admission or 24-hour ED observation, whereas only a small minority required neurosurgical management. This is an important finding, as it suggests that the diagnostic yield of CT and the yield of major therapeutic intervention are not equivalent in this population [2–4, 6, 7, 9, 13].

The overall prevalence of traumatic intracranial lesions observed in our cohort is consistent with the cautious approach still recommended by contemporary guidelines for antithrombotic-treated patients with mild traumatic brain injury [2–4]. At the same time, our results are in line with recent meta-analyses showing that antiplatelet therapy is associated with an increased risk of immediate intracranial hemorrhage, but not necessarily with a proportional increase in the most severe outcomes such as neurosurgical intervention or death [7–9, 19].

Our exploratory multivariable analysis suggests that trauma-related clinical features may be more informative than demographic characteristics alone in this selected population. Falls above standing height or high-energy falls, other trauma mechanisms, motor deficit at presentation, facial fracture, and temporal impact were all independently associated with traumatic intracranial lesions. These findings are clinically plausible and consistent with previous work showing that neurologic abnormalities and objective signs of head or facial trauma are among the strongest markers of intracranial bleeding risk after mTBI [5, 14–16, 20]. By contrast, age was not independently associated with the outcome after adjustment, and female sex showed only a non-significant trend toward lower odds of lesions.

The sensitivity analysis additionally adjusting for dual versus single antiplatelet therapy showed a borderline association between dual antiplatelet therapy and traumatic intracranial lesions. Although this result does not support definitive conclusions, it is directionally consistent with previous reports suggesting that dual antiplatelet therapy may confer higher hemorrhagic risk than single-agent treatment [6, 8, 9, 19]. Repeat brain imaging was not protocolized in the present study and reflected clinician discretion according to lesion characteristics, clinical evolution, and specialist advice. In contrast, among CT-negative patients, very few underwent hospital admission or 24-hour emergency department observation or repeat brain imaging.

This study has several strengths, including its large two-center design, long inclusion period, and systematic use of initial head CT with radiological review. It also has important limitations. First, its retrospective design exposes it to misclassification and incomplete routine-care documentation. Second, only patients who underwent an initial head CT scan were included, which limits inference to imaged patients and does not allow conclusions regarding antiplatelet-treated patients with mTBI who were not scanned. Third, the long study period may have introduced heterogeneity in local imaging thresholds and management practices over time. Fourth, the primary outcome grouped together lesions subtypes with potentially different clinical relevance, and detailed radiological severity features were not available in a sufficiently standardized retrospective format to derive a post hoc severity classification. In addition, because lesion subtype was based on the index radiology report, we could not reliably distinguish acute subdural hematoma from chronic or acute-on-chronic subdural collections in older adults, particularly in patients with possible prior falls. Finally, several symptoms commonly used in clinical decision rules, such as loss of consciousness, vomiting, amnesia, or headache, were not retained because retrospective ascertainment was considered insufficiently reliable. The exploratory multivariable analysis was not intended to derive or validate a prediction rule and should be interpreted as hypothesis-generating only.

Given the elderly profile of the cohort, the systematic use of initial head CT, and the French tertiary-care emergency department setting, these findings are most generalizable to similar hospital-based populations of older antiplatelet-treated adults undergoing emergency neuroimaging.

## Conclusion

In this large two-center cohort of antiplatelet-treated adults with mild traumatic brain injury, traumatic intracranial lesions were detected in approximately one in seven patients on the initial CT scan, whereas neurosurgical intervention remained uncommon. Trauma-related clinical features, rather than age alone, were independently associated with lesion detection. These findings support continued caution in the initial evaluation of this population, while also highlighting the need for more refined risk stratification strategies focused on clinically meaningful outcomes rather than lesion detection alone.

## Data Availability

The datasets generated and/or analyzed during the current study are not publicly available due to patient privacy and regulatory restrictions but are available from the corresponding author on reasonable request.
